# Improving the Performance of Multi-GNSS Time and Frequency Transfer Using Robust Helmert Variance Component Estimation

**DOI:** 10.3390/s18092878

**Published:** 2018-08-31

**Authors:** Pengfei Zhang, Rui Tu, Yuping Gao, Rui Zhang, Na Liu

**Affiliations:** 1 National Time Service Center, Chinese Academy of Sciences, Shu Yuan Road, Xi’an 710600, China; zhangpengfei@ntsc.ac.cn (P.Z.); gaoyp@ntsc.ac.cn (Y.G.); zhangrui@ntsc.ac.cn (R.Z.); liuna@ntsc.ac.cn (N.L.); 2 Key Laboratory of Time and Frequency Primary Standards, Chinese Academy of Sciences, Xi’an 710600, China; 3 University of Chinese Academy of Sciences, Yu Quan Road, Beijing 100049, China; 4 Key Laboratory of Precision Navigation Positioning and Timing Technology, Chinese Academy of Sciences, Xi’an 710600, China

**Keywords:** time and frequency transfer, multi-GNSS, robust estimation, Helmert variance component

## Abstract

The combination of multiple Global Navigation Satellite Systems (GNSSs) may improve the performance of time and frequency transfers by increasing the number of available satellites and improving the time dilution of precision. However, the receiver clock estimation is easily affected by the inappropriate weight of multi-GNSSs due to the different characteristics of individual GNSS signals as well as the outliers from observations. Thus, we utilised a robust Helmert variance component estimation (RVCE) approach to determine the appropriate weights of different GNSS observations, and to control for the influence of outliers in these observation in multi-GNSS time and frequency transfer. In order to validate the effectiveness of this approach, four time links were employed. Compared to traditional solutions, the mean improvement of smoothed residuals is 3.43% using the RVCE approach. With respect to the frequency stability of the time links, the RVCE solution outperforms the traditional solution, particularly in the short-term, and the mean improvement is markedly high at 14.89%.

## 1. Introduction

Since the Global Positioning System (GPS) was first utilized for precise time transfer [1], it has increasingly been applied in time frequency dissemination. From initial common-view (CV) and all-in-view (AV) techniques [2,3], which use only pseudo-range measurements, to the development of carrier-phase (CP) techniques that combine carrier phase observation [4], the accuracy of GPS time transfer has improved significantly. While earlier studies employing CV and AV techniques have recently been re-evaluated, research on GLObal Navigation Satellite System (GLONASS) time transfer has also increasingly been undertaken in those years [5,6]. Recently, other satellite systems have also been well developed, and this has provided a rich resource of Global Navigation Satellite System (GNSS) signals for time and frequency transfer. The European Union’s Galileo satellite system, which was initiated by the European Space Agency (ESA) and the European Commission in 2011 offers one promising E5 signal component with low noise in the centimeter range, and great potential to improve time transfer performance [7,8]. The BeiDou Navigation Satellite System (BDS) of China has been in continuous service since 27 December 2012, and the application of its time transfer has also been investigated [9,10].

Given that combining multi-constellation GNSS can increase the number of available satellites for data retrieval, doing so can improve the redundancy of the receiver clock parameter. While multi-GNSS time transfer has been investigated [11,12,13], such research has primarily focused on combining GPS and GLONASS. As the GLONASS satellites emit signals on individual frequencies via a frequency division multiple access (FDMA) design, the effect of inter-frequency biases (IFBs) [12] and differential code-phase biases (DCPBs) [14] for GLONASS is limited, allowing for the full exploitation of the combination potential of these satellites for precise time and frequency transfer. With the development of precise multi-GNSS satellite orbit and clock products, all satellites within the network use the same time scale for different GNSS products [15,16,17]. This provides the opportunity to combine the three code-division multiple access (CDMA) satellite constellations (i.e., GPS, Galileo and BDS) for precise time and frequency transfer. By increasing the number of available satellites and improving the time dilution of precision, the time transfer solution was determined by combining the three CDMA GNSS constellations, which outperformed single GNSS solutions [18]. However, the quality of orbit and satellite clock products and pseudo-range observation accuracy of Galileo and BDS differ from those of GPS [19], and it is therefore inappropriate to treat them equally as has been done in previous studies [18]. Meanwhile, the pseudo-range observation is an important measurement in time and frequency transfers as it carries temporal information [20,21]. However, outliers are often found in these observations owing to the environments surrounding the stations [22,23], which substantially affect time transfer performance.

In this study, a robust Helmert variance component estimation (RVCE) was introduced when using combined multi-GNSS for precise time and frequency transfer estimates based on a CP technique. This was employed to allocate the adaptive weight for different GNSSs and to suppress the influence of outliers based on their adjustment residuals. Five GNSS stations equipped with external time and frequency references, and which were capable of tracking three GNSS constellations, formed four time links that were processed. The performance of the CP technique with three GNSS observations using the RVCE approach was assessed in terms of both time transfer noise level as well as time and frequency stability [13]. The structure of this manuscript is organized as follows: Section 2 describes the observation equation of the time and frequency transfer based on multi-GNSS. The robust approach based on the Helmert variance component estimation (VCE) is discussed in Section 3, and the implementation procedure is also summarized. Thereafter, Section 4 describes the experiment that was used to assess performance, and the comparison and validation with the traditional solutions are also presented. The summary and conclusions are given in Section 5.

## 2. Observation Equation of Multi-GNSS CP Time Transfer

In general, the mathematic model of time transfer based on a CP technique is the same as in the precise point positioning (PPP) model. The ionosphere-free combination for individual GNSSs has typically been utilized for dual-frequency carrier phase and pseudo-range observations to eliminate the first-order effects of the ionosphere. The observation equations can be written as [21,24]:

(1) $$\begin{array}{c} P=\rho +c\cdot (dt_{r}-dt_{s})+T_{trop}+\varepsilon_{P}\\ L=\rho +c\cdot (dt_{r}-dt_{s})+T_{trop}+N_{IF}+\varepsilon_{L} \end{array}$$

where, P and *L* are the ionosphere-free combinations of carrier phase and pseudo-range. The geometric distance is denoted as ρ, c is the speed of light, $T_{trop}$ is the tropospheric delay, $N_{IF}$ is the phase ambiguity of the ionosphere-free (IF) combination in metres, $\varepsilon_{P}$ and $\varepsilon_{L}$ are the measurement noise and multipath error for pseudo-range and carrier phase, respectively, $dt_{s}$ is the satellite clock offset and $dt_{r}$ is the receiver clock offset, which is the difference between the external time frequency reference and the GNSS time scale. It is important to note that the phase centre offset and variation, tidal loading, ocean tides, phase wind-up and relativistic delay must be corrected according to the existing models [24], although they are not explicitly expressed in Equation (1).

In the combined GPS/BDS/Galileo time and frequency transfer based on the CP technique, there are three receiver clock offsets that represent differences between the common external time frequency reference and their corresponding system time scales. When the GPS time scale (GPST) is selected as a unique reference time scale to provide the common receiver clock offset based on three CDMA GNSSs, additional inter-system bias (ISB) parameters in BDS and Galileo are introduced, which also refer to inter-system receiver hardware bias. The formula may be expressed as:

(2) $$\left\{ \begin{array}{l} ISB_{sys}^{GC}=dt_{r}^{C}-dt_{r}^{G}\\ ISB_{sys}^{GE}=dt_{r}^{E}-dt_{r}^{G} \end{array}\right.$$

The combined observation equation using GPS/BDS/Galileo measurements can therefore be expressed as [18]:

(3) $$\left\{ \begin{array}{l} P_{i}^{G}=\rho_{i}^{G}+c\cdot (dt_{r}^{G}-dt_{i,s}^{G})+T_{trop}+\varepsilon_{i,P}^{G}\\ L_{i}^{G}=\rho_{i}^{G}+c\cdot (dt_{r}^{G}-dt_{i,s}^{G})+T_{trop}+N^{G}+\varepsilon_{i,P}^{G}\\ P_{i}^{C}=\rho_{i}^{C}+c\cdot (dt_{r}^{G}+ISB_{sys}^{GC}-dt_{i,s}^{C})+T_{trop}+\varepsilon_{i,P}^{C}\\ L_{i}^{C}=\rho_{i}^{C}+c\cdot (dt_{r}^{G}+ISB_{sys}^{GC}-dt_{i,s}^{C})+T_{trop}+N^{C}+\varepsilon_{i,P}^{C}\\ P_{i}^{E}=\rho_{i}^{E}+c\cdot (dt_{r}^{G}+ISB_{sys}^{GE}-dt_{i,s}^{E})+T_{trop}+\varepsilon_{i,P}^{E}\\ L_{i}^{E}=\rho_{i}^{E}+c\cdot (dt_{r}^{G}+ISB_{sys}^{GE}-dt_{i,s}^{E})+T_{trop}+N^{E}+\varepsilon_{i,P}^{E} \end{array}\right.$$

where the superscripts G, C, and E represent the GPS, BDS, and Galileo satellite systems, respectively. The subscript i denotes a single satellite. It is notable that the $dt_{r}^{G}$ becomes a parameter that is jointly determined by GPS, BDS and Galileo satellite systems measurements, which are the quantities of interest when applying precise time and frequency transfer. When the two remote stations m and n equipped with different time and frequency references $t_{m}$ and $t_{n}$, are used for time and frequency transfer, the common GPST plays a role in the intermediate quantity to achieve time and frequency transfer progress; the corresponding formula can be expressed as follows:

(4) $$\Delta T_{m,n}=dt_{r}^{G}\left(m\right)-dt_{r}^{G}\left(n\right)=\left(t_{m}-GPST\right)-\left(t_{n}-GPST\right)=t_{m}-t_{n}$$

Equation (4) indicates that the receiver clock offset is a parameter of interest in time and frequency transfer. After the combined observation Equation (3) has been transformed and linearized, one least squares (LS) estimator is employed to determine the unknown parameters in the simplified Equation (5):

(5) $$X={\left(A^{T}PA\right)}^{-1}A^{T}PL$$

where A is the design matrix of the parameters to be estimated, L denotes the observed value minus the computed value, and P is the corresponding weight matrix. The parameter vector X may be estimated from the expression:

(6) $$X=\left[x,y,z,dt_{r}^{G},ISB_{sys}^{GC},ISB_{sys}^{GE},T_{trop},N^{G},N^{C},N^{E}\right]$$

## 3. Robust Helmert Variance Component Estimation (RVCE)

In Equation (5), the weight matrix is the combined weight of the three GNSSs. As the observation qualities of individual GNSSs are not the same due to the different signal structures, frequencies, accuracies of satellite orbit and clock products, signal-to-noise ratios and signal transmission paths, neither individual GNSSs nor the individual satellites should be treated equally [15,19]. Although the existing LS-VCE method can be employed to allocate the weight of different kinds of observation [25], it has lack of robust function. Moreover, in the area of precise time and frequency transfer based on multi-GNSSs, outliers inevitably exist in observations [26,27]. These outliers will further distort the results of receiver clock estimation, and do not reasonably represent the characteristics of the time and frequency references. Therefore, a robust approach based on the Helmert VCE is introduced in this study to allocate the appropriate weights and to control for the influence of outliers, which are generally referred to as the posterior measurement residuals.

The error equation of the three combined GNSSs for time and frequency can be written as:

(7) $$V=A\cdot \hat{X}-L=\left[\begin{array}{c} V_{G}\\ V_{C}\\ V_{E} \end{array}\right]=\left[\begin{array}{c} A_{G}\\ A_{C}\\ A_{E} \end{array}\right]\cdot \hat{X}-\left[\begin{array}{c} L_{G}\\ L_{C}\\ L_{E} \end{array}\right]$$

where, V denotes the combination posterior measurement residuals, which can be further written as the residuals of the individual systems (i.e., GPS, BDS and Galileo) and $\hat{X}$ is the estimated vector of unknown parameters. Helmert VCE (i.e., posterior variance stochastic model estimation) is based on the residuals derived from the initial inappropriate weights (i.e., prior equal weights) for the observations. In the practical application of combining the three GNSSs for time and frequency transfer, the range of the GNSS residual values may differ due to their different systematic errors. The individual residuals therefore must be unified to a common range using their corresponding variances and cofactors, the formula for this unification is given as:

(8) $$\tilde{V}\left(i\right)=\frac{V\left(i\right)}{\sigma_{0}^{2}\sqrt{Q\left(i\right)}}$$

where $\tilde{V}$ denotes the standard residual matrix. The unit weight variance is $\sigma_{0}^{2}$ and Q is the corresponding cofactor, which can be determined using Equations (9) and (10), respectively:

(9) $$\sigma_{0}^{2}=\frac{V^{T}PV}{n-m}$$

(10) $$Q=P^{-1}-AN^{-1}A^{T}$$

where, n is the number of available GNSS satellites, m denotes the estimated parameter number and N is the matrix of the normal equation. Therefore, according to the Helmert VCE theory, the estimated variance components can be determined using the following formulae:

(11) $$\left[\begin{array}{c} {\hat{\sigma }}_{0,G}^{2}\\ {\hat{\sigma }}_{0,C}^{2}\\ {\hat{\sigma }}_{0,E}^{2} \end{array}\right]={\left[\begin{array}{ccc} s_{G,G} & s_{G,C} & s_{G,E}\\ s_{G,C} & s_{C,C} & s_{C,E}\\ s_{G,E} & s_{C,E} & s_{E,E} \end{array}\right]}^{-1}\left[\begin{array}{c} {\tilde{V}}_{G}^{T}P_{G}{\tilde{V}}_{G}\\ {\tilde{V}}_{C}^{T}P_{C}{\tilde{V}}_{C}\\ {\tilde{V}}_{E}^{T}P_{E}{\tilde{V}}_{E} \end{array}\right]$$

(12) $$\begin{array}{r} s_{k,k}=n_{k}-2tr\left(N^{-1}N_{k}\right)+tr\left(N^{-1}N_{k}N^{-1}N_{k}\right)\\ s_{k,j}=n_{k}-tr\left(N^{-1}N_{k}N^{-1}N_{j}\right)\\ N=A^{T}PA \end{array}\}$$

where, tr is the trace operation for matrix. The initial weights for three GNSSs are typically inappropriate, and the corresponding variance components ${\hat{\sigma }}_{0,G}^{2}$, ${\hat{\sigma }}_{0,C}^{2}$, and ${\hat{\sigma }}_{0,E}^{2}$ are also unequal. The relationship between the redefined weight matrix and the estimated components is as follows:

(13) $${\hat{P}}_{k}=\frac{c_{0}}{\sigma_{0,k}^{2}}P_{k}$$

where $\sigma_{0,k}^{2}$ is the estimated variance component for the three GNSSs, $c_{0}$ is constant and usually selected from one of the estimated variance components (i.e., $\sigma_{0,k}^{2}$), and $P_{k}$ is the initial weight for the three GNSSs. With this new weight matrix, the residuals of the three GNSSs are recalculated until the unit weight variance components of the three systems are equal or have ratios approximately equal to one.

The Helmert VCE is able to obtain an appropriate posterior weight, however, it is not robust to the influence of outliers. In the domains of time and frequency transfer, outliers are inevitably encountered due to code observation noise, equipment failures, and external interference factors [27,28], which can all result in failure of the estimated parameters to reasonably represent the characteristics of the atomic clocks. Therefore, robust estimation [29,30] is introduced to reduce the influence of outliers when combining three GNSSs for time and frequency transfer, this has also been widely applied in geodesy [31,32].

On the basis of the robust estimation theory, the LSQ estimation can be written as follows:

(14) $$\bar{X}={\left(A^{T}\;\bar{P}A\right)}^{-1}\;A^{T}\bar{P}L$$

where, $\bar{P}$ denotes an equivalent weight matrix, which is derived from the distribution of individual GNSS residuals and their equivalent weight function. In this study, the equivalent weight function can be defined by the Institute of Geodesy and Geophysics 3 (IGG3) scheme [33,34], which is constructed with three segments [32,35] based on the following formula:

(15) $$\bar{P_{i}}=\{\begin{array}{cc} P_{i} & ,\left|{\tilde{V}}_{k}{}_{i}\right|\leq k_{0}\\ P_{i}\frac{k_{0}}{\left|{\tilde{V}}_{k}{}_{i}\right|}{\left(\frac{k_{1}-\left|{\tilde{V}}_{k}{}_{i}\right|}{k_{1}-k_{0}}\right)}^{2} & ,k_{0}<\left|{\tilde{V}}_{k}{}_{i}\right|\leq k_{1}\\ 0 & ,\left|{\tilde{V}}_{k}{}_{i}\right|>k_{1} \end{array}$$

where $k_{0}$ and $k_{1}$ denote threshold constants depending on the actual distribution, which are typically chosen as $k_{0}\in \left[1.0,\;1.5\right]$ and $k_{1}\in \left[2.0,\;8.0\right]$, respectively.

## 4. Implementation of Multi-GNSS Time and Frequency Transfer using RVCE

**Figure 1:**
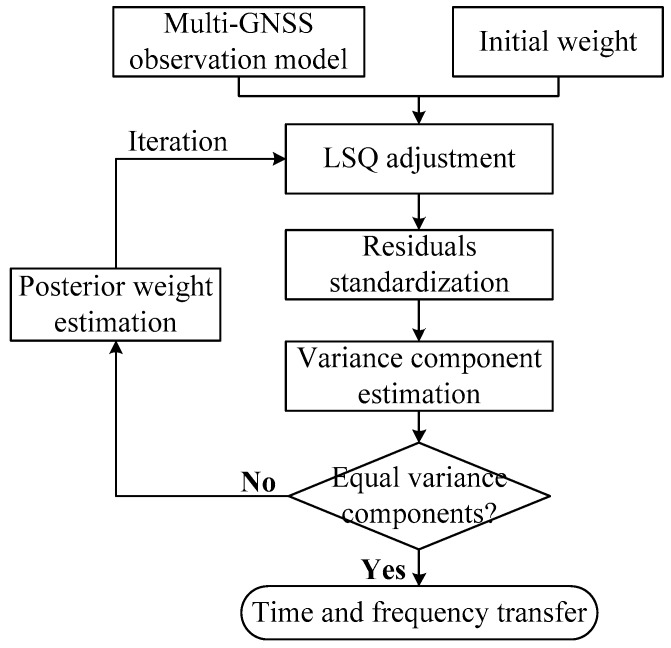
Primary implementation procedure using robust Helmert variance component estimation (RVCE) for time and frequency transfer.

Details of the four-step data processing procedure for time and frequency transfer using a RVCE algorithm are shown in Figure 1. First, when combining the three CDMA GNSSs for time and frequency transfer, the observation model can be formed according to Equation (3), and the receiver clock offset is our parameter of interest. Meanwhile, the initial weights of the three GNSSs are temporarily set as equal. The parameters in Equation (6) are then estimated using LSQ adjustment. The residuals of all satellites can be derived according to Equation (7), and may be further standardized by Equation (8). Next, the three variance components are estimated according to Equation (11). It is worth noting that when the robust function is employed, the weighs matrices for the three GNSSs in Equation (11) must be determined by their equivalent weight functions and standardized residuals before the robust variance components may be acquired. Finally, whether the three weight variance components are equal or not must be judged. If equal, the previous weights from the second step are output and used to estimate receiver clock parameters based on Equation (5), thereafter, the progress of time and frequency transfer is completed using Equation (4). If the weight variance components are not equal, the posterior weights are determined based on Equation (11), and then used to begin the next loop in LSQ adjustment until the three weight variance components are equal.

## 5. Experimental Design

**Figure 2:**
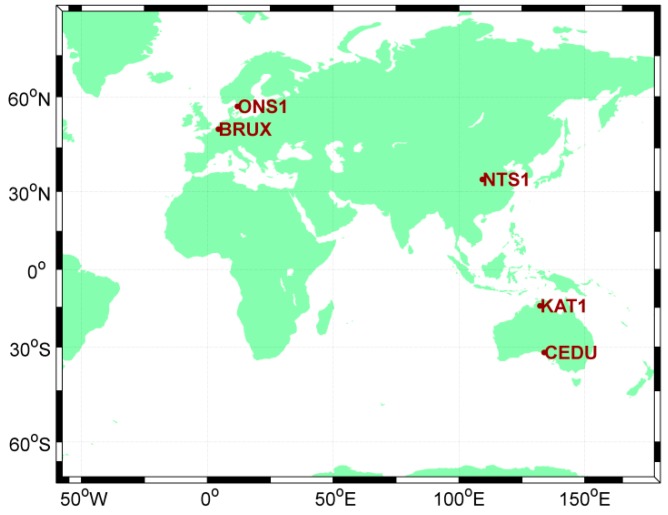
Layouts of multi-GNSS stations, capable of tracking GPS, BDS and Galileo constellations.

In order to assess the effectiveness of the RVCE approach outlined above, an experiment considering the time link length was devised, the data spanned from MJD 57981 to MJD 57986. Five GNSS stations equipped with stable external time and frequency signals were examined for this experiment. These stations are all able to track the GPS, BDS and Galileo satellite constellations (Figure 2). Two of the stations (NTS1 and BRUX) are located at two time laboratories, the National Time Service Centre (NTSC), and Observatoire Royal de Belgique (ORB)), which are connected with the UTC (NTSC) and UTC (ORB) time and frequency signals, respectively. Another three stations are equipped with external H-maser references. The attributes of these five stations are given in Table 1.

When the time and frequency transfer was employed, the time link was formed between the two stations, which was the primary unit in the area of time and frequency transfer. We therefore established four time links using the five stations with geodetic distances from 883.7 to 7537.5 km, and each link has at least one UTC signal provided by UTC (NTSC) or UTC (ORB), which are summarized in Table 2.

## 6. Results and Discussion

The performance of time and frequency transfer determined by a CP technique with a RVCE approach (i.e., a RVCE scheme) was compared with the performance of the traditional approach that treats the three GNSSs equally (i.e., the raw scheme). In data processing, the dual-frequency observations (L1/L2 for GPS, B1/B2 for BDS and E1/E5a for Galileo) are used to establish their corresponding ionosphere-free combinations for eliminating the first-order effects of the ionosphere. The combinations for pseudo-range and carrier phase measurement are also called P3 and L3 observation in the area of time and transfer [36,37,38]. The cycle slips of carrier phase are detected by the geometry-free (GF) and Melbourne-Wübbena combination [39], which are performed according to the signal frequency of each satellite system. The satellites with elevations less than 7° are eliminated. Multi-system precise satellite orbit and clock products are obtained from the Multi-GNSS Experiment (MGEX) Center for Orbit Determination in Europe (CODE), in which system time refers to the GPS system scale [40]. The hydrostatic component of the tropospheric delay is corrected using a Saastamoinen model, and the residual non-hydrostatic component is estimated as a random-walk. It is noteworthy that the parameter of interest with respect to the receiver clock offset is estimated by combining pseudo-range and carrier phase observations following a white noise stochastic process. In brief, the receiver clock offsets at adjacent epochs are uncorrelated, and are utilized based on the characteristics of a free-running atomic clock. The details of the observation models and data processing strategies are provided in Table 3. With respect to the RVCE, we used the ratios between pairs of the three estimated variance components as the threshold to control the number of iterations in the experiment. If the ratio value was within the range of 0.9 to 1.1, the iteration was stopped. The unique receiver clock offset parameter was then estimated, and the results of the time transfer were further derived using Equation (4). 

**Figure 3:**
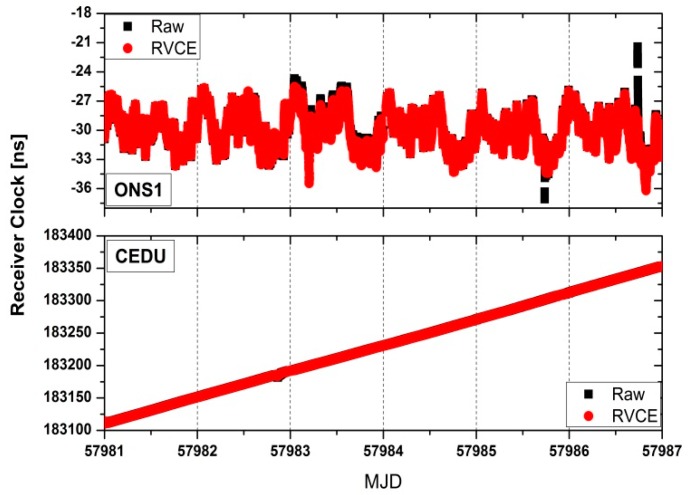
Receiver clock time series for station ONS1 and CEDU with and without the RVCE approach.

Figure 3 preliminarily presents a typical example of receiver clock time series at two stations with and without the RVCE approach. It can be noted the receiver clock value has unobvious discrepancy for two approaches apart from a few epochs. Considering that the time link is the primary analysis unit in practical work of time and frequency transfer, we therefore pay more attention to it in following text.

**Figure 4:**
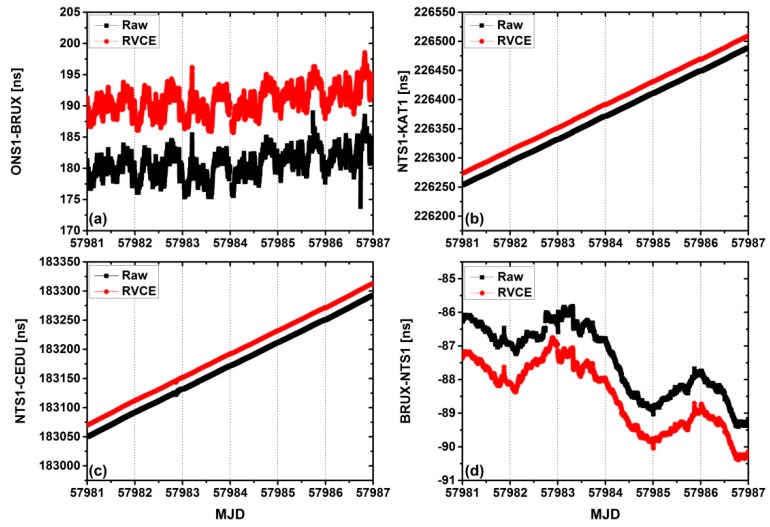
Results of time transfers determined by a carrier-phase (CP) technique with and without the RVCE approach for the four time links. (**a**) is for ONS1-BRUX, (**b**) is for NTS1-KAT1, (**c**) is for NTS1-CEDU and (**d**) is for BRUX-NTS1.

The results of the time transfer as determined by the multi-GNSS CP technique using the raw and RVCE schemes for all four time links are shown in Figure 4. The RVCE solutions are shown in red and the raw solutions are shown in black. To ensure the clarity of our purpose, the solutions from the RVCE scheme have been translated (10 ns for ONS1-BRUX, 20 ns for NTS1-KAT1, 20 ns for NTS1-CEDU, and −2 ns for BRUX-NTS1).

**Figure 5:**
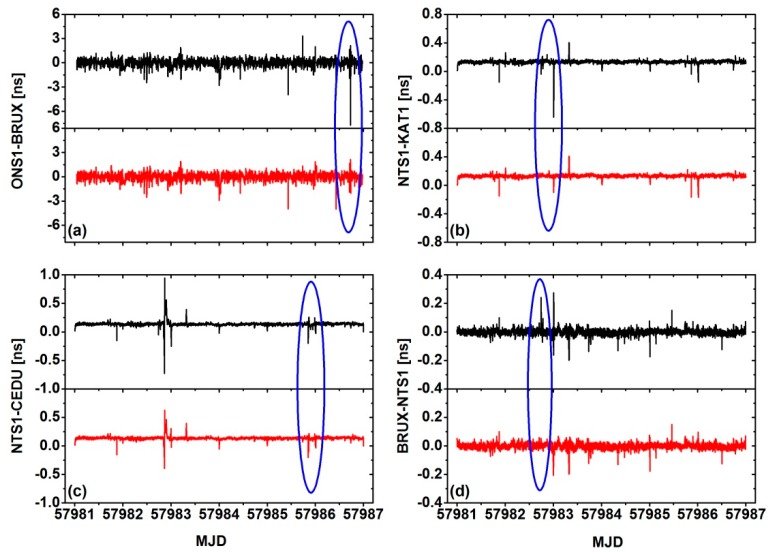
Smoothing residuals of the results with and without the RVCE approach for the four time links. (**a**) is for ONS1-BRUX, (**b**) is for NTS1-KAT1, (**c**) is for NTS1-CEDU and (**d**) is for BRUX-NTS1.

In the time link of ONS1-BRUX, it can be found that the RVCE scheme has effectively resisted some outliers. Due to the external time and frequency references at the stations, CEDU and KTA1 have not been controlled, while the trend terms are obvious, the performances are difficult to compare. Therefore, the residuals and the root mean square (RMS) value were compared, and the smoothed result is always used because it maintains both low signal noise and the variable trend of the time transfer results. The Kalman smoothing approach was employed in our experiment. Figure 5 shows the residuals of the two different schemes for the four time links, the black line is the traditional solution and the red line is RVCE solution. It can be clearly seen that the RVCE can resist more outliers when compared to the traditional approach. Table 4 presents the corresponding RMS for the time links. The performance of the RVCE solution is slightly improved relative to the raw solution, which ranges from 0.75% to 5.48%, with a mean of 3.41%.

**Figure 6:**
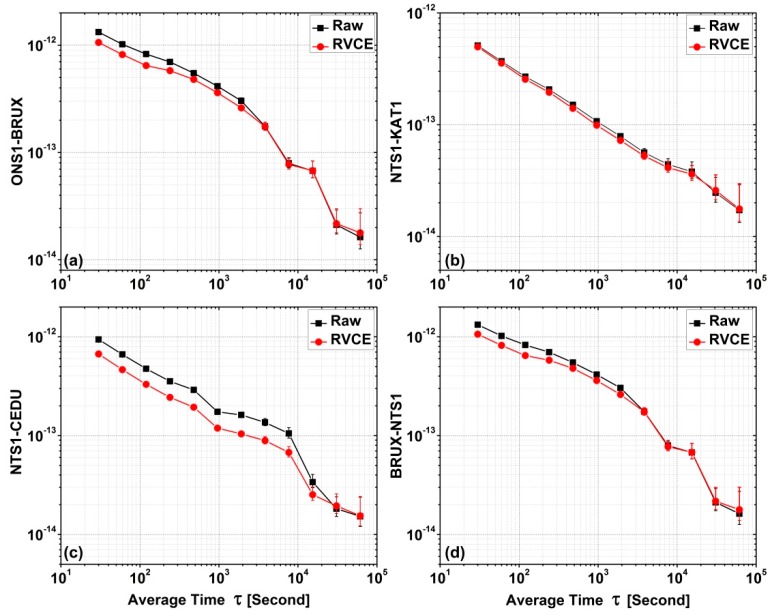
Comparison of Allan deviation (ADEV) for the four time links determined with and without RVCE. (**a**) is for ONS1-BRUX, (**b**) is for NTS1-KAT1, (**c**) is for NTS1-CEDU and (**d**) is for BRUX-NTS1.

**Figure 7:**
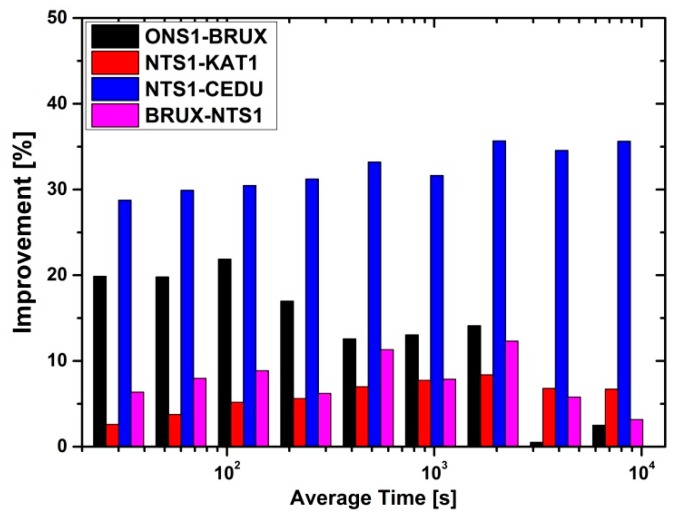
Improvement to stability (%) of RVCE solutions over the raw solutions within 10,000 s.

In the domains of time and frequency, the frequency stability is an important index for time links, particularly for short term when using the GNSS technique [42]. Here, the Allan deviation (ADEV) of the time transfer solutions for two different scenarios is obtained for the four time links (Figure 6) and the corresponding ADEV values are given in Table 5. It is clear that the stability of the RVCE solution is greater than that of the raw solution at different average time (AT) τ, particularly when AT are within 10,000 s. Because the number of available data points used for computing ADEV dramatically decreases when τ is greater than 10,000 s, the corresponding measurement errors gradually increase. The error bars at different τ in the Figure 6 also can be agreed with this principle. Therefore, the results that the τ is less than 10,000 s are pay more attention and corresponding improvement for these four time links are summarized in Figure 7. It can be noted that the improvement range relative to the raw solutions differ among the four links. The mean improvement to the ONS1-BRUX link is 13.48%, while improvements are 5.97% for the NTS1-KAT1 link, 32.34% for the NTS1-CEDU link and 7.76% for the BRUX-NTS1 link. The mean value for all four time links is 14.89%.

## 7. Conclusions

In this study, we analyzed the combined multi-GNSS for CP time and frequency transfer using an RVCE approach, and the corresponding observation model was considered. The RVCE approach and data processing procedure were also detailed. In order to evaluate the performance of the RVCE, four time transfer links with external time and frequency references were computed with and without RVCE. Because that receiver clock estimation is easily affect by the inappropriate weights of multi-GNSSs and outliers from observations, which is closely related to the performance of precise time and frequency transfer. The RVCE approach used in this study is able to determine the observed weight for each GNSS and resist the influence of outliers and shows certain superiority to the raw solution. The smoothed residuals of this method are superior to those of the traditional multi-GNSS CP technique, and the corresponding RMS values were slightly improved for the four time links using RVCE, the range is 5.48% for ONS1-BRUX, 0.75% for NTS1-KAT1, 2.13% for NTS1-CEDU and 5.26% for BRUX-NTS1, respectively. With respect to the frequency stability of time links, the improvements over the short-term within 10,000 s are obvious. The average range is 13.48% for ONS1-BRUX link, 5.97% for NTS1-KAT1 link, 32.34% for NTS1-CEDU link, 7.76% for BRUX-NTS1 link. Therefore, combining multi-GNSS for CP time and frequency transfers using an RVCE approach can improve the validity of the solution within a certain precision range for different time links, particularly for observations that include outliers.

## Figures and Tables

**Table 1:** Attributes of stations used in this study.

Site Name	GNSS Receiver	Antenna	Frequency Standard
NTS1	SEPT POLARX4TR	SEPCHOKE_MC	UTC (NTSC)
BRUX	SEPT POLARX4TR	JAVRINGANT_DM	UTC (ORB)
KAT1	SEPT POLARX5	LEIAR25.R3	EXTERNAL H-MASER
CEDU	SEPT POLARX5	AOAD/M_T	EXTERNAL H-MASER
ONS1	TRIMBLE NETR9	LEIAR25.R3	EXTERNAL H-MASER

**Table 2:** Time links formed and their geodetic distances.

Time Link Name	Geodetic Distance (km)
ONS1-BRUX	883.7
NTS1-KAT1	5704.2
NTS1-CEDU	7294.5
BRUX-NTS1	7537.5

**Table 3:** Observation models and multi-system RINEX data processing strategies.

Items	Models and Strategies
Observations	Undifferenced carrier phase and code observation
Signal selection	GPS:L1/L2; BeiDou: B1/B2; Galileo: E1/E5a
Satellite orbit and clock	Using the precise satellite products from CODE [40]
Satellite antenna phase center	Corrected using MGEX value
Initial weight between code and phase	0.0001:1
Ionosphere	Eliminated by ionosphere-free combination [36,37,38]
Tropospheric delay	Initial model + random-walk process
Tropospheric mapping function	Neill mapping function (NMF) [41]
Elevation cutoff	7°
Sampling rate	30 s
Observation weight	Elevation dependent weight
Estimator	LS in sequential mode
Receiver clock offset	Estimated with white noise
ISB	Estimated with epoch-wise variable method
Phase wind-up effect	Model corrected

**Table 4:** Improvement (%) in RMS of the result smoothing in two scenarios for the time links.

Time Link Name	Raw (ns)	RVCE (ns)	Improvement (%)
ONS1-BRUX	0.420	0.397	5.48
NTS1-KAT1	0.133	0.132	0.75
NTS1-CEDU	0.141	0.138	2.13
BRUX-NTS1	0.019	0.018	5.26

**Table 5:** ADEV values of the four time links determined with and without RVCE.

τ (seconds)	Time Link Name							
	ONS1-BRUX		NTS1-KAT1		NTS1-CEDU		BRUX-NTS1	
	Raw	RVCE	Raw	RVCE	Raw	RVCE	Raw	RVCE
30	1.32 × 10^−12^	1.06 × 10^−12^	5.10 × 10^−13^	4.97 × 10^−13^	9.38 × 10^−13^	6.69 × 10^−13^	4.08 × 10^−13^	3.82 × 10^−13^
60	1.02 × 10^−12^	8.17 × 10^−13^	3.69 × 10^−13^	3.55 × 10^−13^	6.63 × 10^−13^	4.65 × 10^−13^	2.58 × 10^−13^	2.37 × 10^−13^
120	8.27 × 10^−13^	6.46 × 10^−13^	2.69 × 10^−13^	2.55 × 10^−13^	4.75 × 10^−13^	3.30 × 10^−13^	1.60 × 10^−13^	1.46 × 10^−13^
240	6.98 × 10^−13^	5.79 × 10^−13^	2.06 × 10^−13^	1.95 × 10^−13^	3.54 × 10^−13^	2.44 × 10^−13^	1.03 × 10^−13^	9.68 × 10^−14^
480	5.48 × 10^−13^	4.79 × 10^−13^	1.50 × 10^−13^	1.40 × 10^−13^	2.90 × 10^−13^	1.94 × 10^−13^	8.00 × 10^−14^	7.10 × 10^−14^
960	4.14 × 10^−13^	3.60 × 10^−13^	1.07 × 10^−13^	9.90 × 10^−14^	1.74 × 10^−13^	1.19 × 10^−13^	5.95 × 10^−14^	5.48 × 10^−14^
1920	3.03 × 10^−13^	2.60 × 10^−13^	7.88 × 10^−14^	7.22 × 10^−14^	1.62 × 10^−13^	1.04 × 10^−13^	4.33 × 10^−14^	3.80 × 10^−14^
3840	1.75 × 10^−13^	1.74 × 10^−13^	5.63 × 10^−14^	5.25 × 10^−14^	1.36 × 10^−13^	8.91 × 10^−14^	2.83 × 10^−14^	2.67 × 10^−14^
7680	7.92 × 10^−14^	7.72 × 10^−14^	4.42 × 10^−14^	4.13 × 10^−14^	1.05 × 10^−13^	6.76 × 10^−14^	2.26 × 10^−14^	2.19 × 10^−14^
15,360	6.74 × 10^−14^	6.77 × 10^−14^	3.80 × 10^−14^	3.61 × 10^−14^	3.39 × 10^−14^	2.53 × 10^−14^	1.90 × 10^−14^	1.57 × 10^−14^
30,720	2.11 × 10^−14^	2.17 × 10^−14^	2.46 × 10^−14^	2.58 × 10^−14^	1.82 × 10^−14^	1.95 × 10^−14^	1.22 × 10^−14^	1.19 × 10^−14^
61,440	1.62 × 10^−14^	1.78 × 10^−14^	1.72 × 10^−14^	1.76 × 10^−14^	1.52 × 10^−14^	1.55 × 10^−14^	1.56 × 10^−14^	1.37 × 10^−14^
